# Four polymorphic structures of a symmetric azo dye

**DOI:** 10.1039/d5ce01159k

**Published:** 2026-02-09

**Authors:** Max T. Hill, Mark A. Birch-Machin, Jonathan D. Sellars, Paul G. Waddell

**Affiliations:** a Biosciences Institute, Faculty of Medical Sciences, Newcastle University Newcastle upon Tyne NE1 7RU UK; b School of Pharmacy, Faculty of Medical Sciences, Newcastle University Newcastle upon Tyne NE1 7RU UK; c Translational and Clinical Research Institute, Faculty of Medical Sciences, Newcastle University Newcastle upon Tyne NE1 7RU UK; d School of Natural and Environmental Sciences, Bedson Building, Newcastle University Newcastle upon Tyne NE1 7RU UK paul.waddell@ncl.ac.uk

## Abstract

The synthesis and structural characterisation of a symmetric azo dye, ethyl azocinnamate, *via* single crystal X-ray diffraction led to the discovery of four polymorphic forms. The crystals and their structures were analysed in terms of morphology, conformation, intermolecular interactions and overall crystal packing. This analysis provides insight into their properties, formation and stability. Two distinct conformations were observed, with two representative structures of each. The structures are layered, and were observed to grow as flat, planar crystals with the exception of one, the prismatic habit of which could be attributed to the strong inter-layer interactions unique to this form. The elastic properties of one of the polymorphs was linked to columns of π⋯π interactions along one direction, a feature absent in the more rigid forms. One polymorph with *Z*′ > 1, which crystallises in the space group *P*2_1_, is analysed in terms of approximate symmetry and is found to be a distorted *P*2_1_/*c* structure.

## Abstract 2

The synthesis and structural characterisation of a symmetric azo dye, ethyl azocinnamate, *via* single crystal X-ray diffraction led to the discovery of four polymorphic forms.

## Graphical abstract

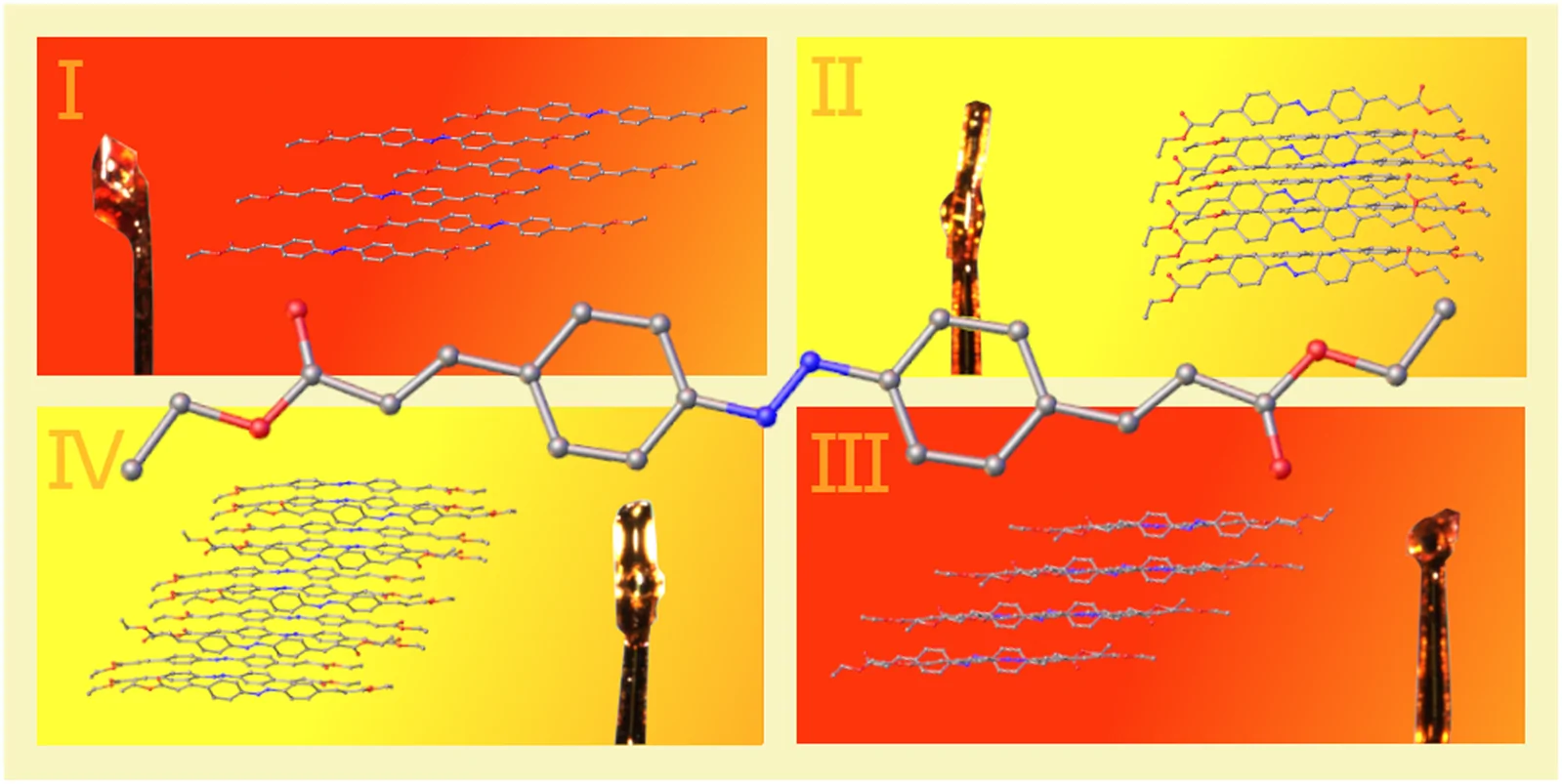

## Introduction

Azo dyes constitute the largest and most diverse class of synthetic colourants in modern chemical, industrial and technological applications. Their predominance reflects both their structural simplicity and the remarkable tunability of the azo chromophore (–N=N–), enabling fine control over colour, solubility, and substrate affinity. More than 60% of all commercial dyes are azo-based, a propensity supported by their straightforward preparation and a wide availability of aromatic precursors.^1^ Industrial usage spans textiles, printing, paper, cosmetics, pharmaceuticals, food technology and advanced functional materials,^2^ giving azo dyes a central position in global colour chemistry.^3^

**Fig. 1:**
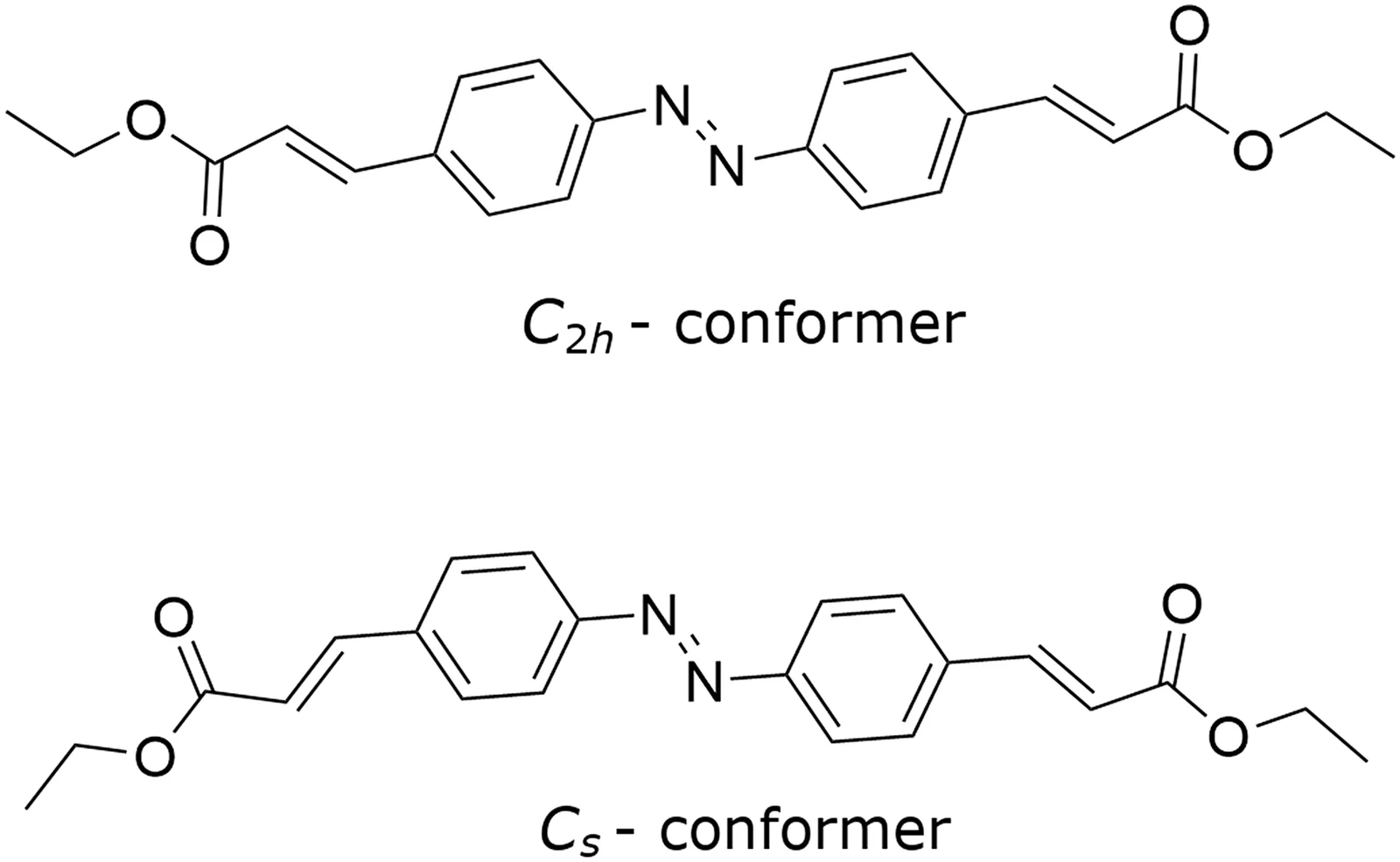
The two conformations of ethyl azocinnamate observed in crystal structures of this compound.

As part of an ongoing programme to identify catechol-based derivatives capable of preventing DNA damage, we undertook the synthesis of a series of sulfonamides designed to explore this chemical space.^4^ During the reaction of ethyl 4-aminocinnamate and 3,4-dimethoxybenzenesulphonyl chloride, an unexpected impurity was observed. Subsequent isolation and characterisation revealed that this species was ethyl azocinnamate (Fig. 1), an azo dye first isolated in trace amounts from the synthesis of triazole compounds from ethyl 4-nitrocinnamate,^5^ formed from ethyl 4-aminocinnamate through an unanticipated side reaction. Crystallisation of the purification of ethyl azocinnamate produced an additional and unforeseen outcome. After only a handful of crystallisation experiments the azo compound yielded four distinct crystalline forms.

Given the importance of the phenomenon of polymorphism,^6,7^ not just in terms of crystallography and structural science, but that the solid-state form of a compound can affect a variety of bulk properties and favour the application of one over the others for specific uses, systematic studies of the structures of polymorphs and how they differ are of use to those in the field of crystal engineering. The discovery of four polymorphs within such a short investigative window is especially notable,^8^ particularly given the absence of any prior structural information for ethyl azocinnamate in the Cambridge Structural Database.^9^

Herein, we report and describe four polymorphs of ethyl azocinnamate, outlining their discovery and structural features in terms of their conformation, intermolecular contacts and packing and how these can be correlated to their properties.

## Experimental

As previously outlined,^4^ ethyl 4-aminocinnamate and 3,4-dimethoxybenzene sulfonyl chloride were reacted to produce the ethyl azocinnamate dye as a by-product in low yield, *δ*_H_ (400 MHz, CDCl_3_) 7.87 (2H, d, *J* 8), 7.66 (1H, d, *J* 16), 7.61 (2H, d, *J* 8), 6.46 (1H, d, *J* 16), 4.23 (2H, q, *J* 7) 1.28 (3H, t, *J* 7); *δ*_C_ (101 MHz, CDCl_3_) 168.6, 153.5, 144.74, 136.9, 129.2, 123.6, 119.1, 61.8, 14.1; *m*/*z* (ES^+^) 379 (MH^+^); HRMS (ES^+^) found MH^+^, 379.1649 (C_22_H_23_N_2_O_4_ requires 379.1652).

Crystals suitable for single crystal X-ray analysis were grown *via* slow evaporation of the solvent from a solution of the compound in ethyl acetate (I, III and IV) or acetonitrile (II). In addition, crystals of the starting materials used in both previously reported syntheses, ethyl 4-aminocinnamate (S1) and ethyl 4-nitroocinnamate (S2), were grown from heptane and dichloromethane/acetone respectively. Single crystal diffraction data were collected on an XtaLAB Synergy-S HyPix-Arc 100 diffractometer using copper radiation (*λ*_CuKα_ = 1.54184 Å) at 150 K using an Oxford Cryosystems CryostreamPlus open-flow N_2_ cooling device. Intensities were corrected for absorption using a multifaceted crystal model created by indexing the faces of the crystal for which data were collected.^10^ Cell refinement, data collection and data reduction were undertaken *via* the software CrysAlisPro.^11^

**Fig. 2:**
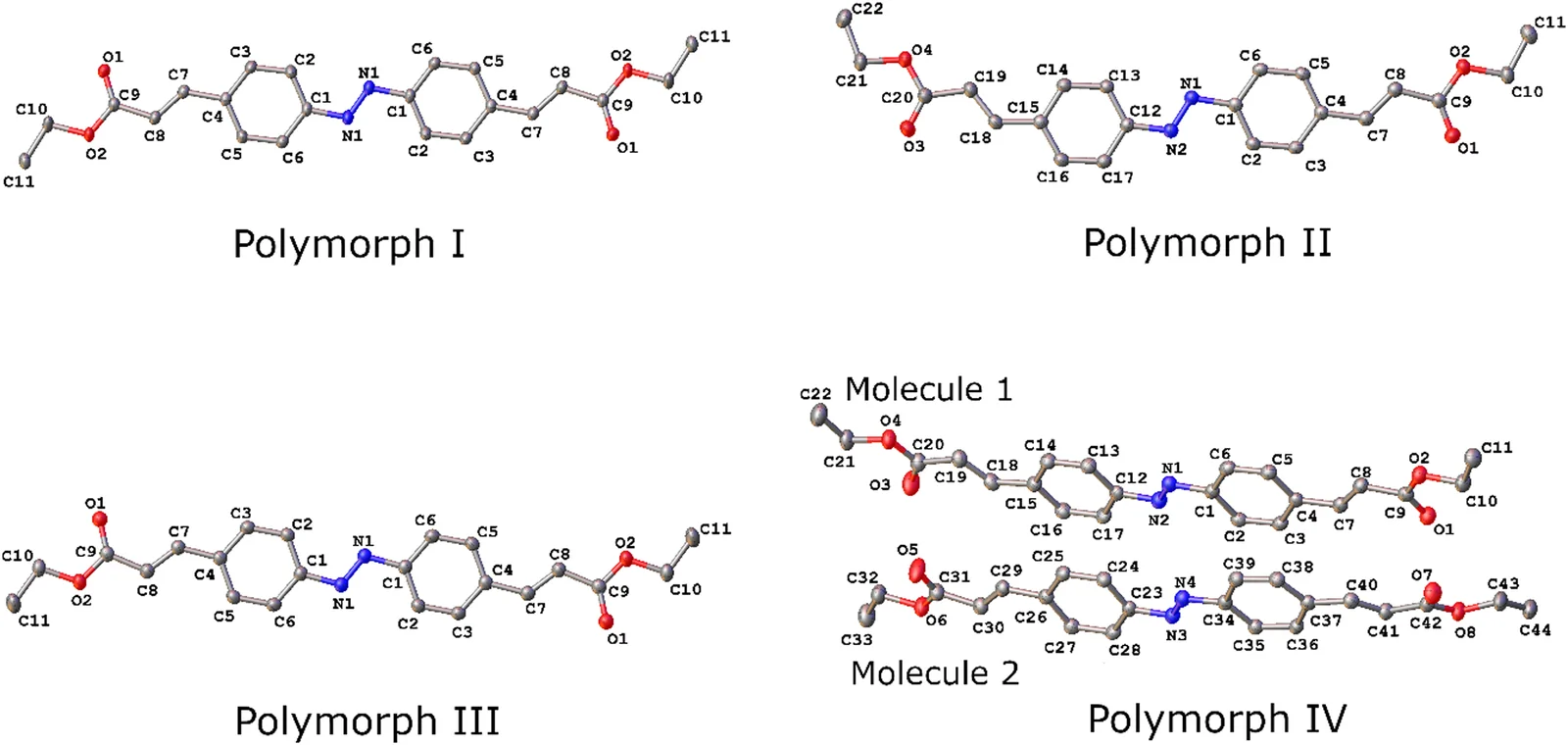
Molecular structure of ethyl azocinnamate in four polymorphic forms with ellipsoids drawn at the 50% probability level. Hydrogen atoms bound to carbon atoms have been omitted for clarity.

All structures were solved using XT^12^ and refined by XL^13^ using the Olex2 interface^14^ (Fig. 2, Table 1). All non-hydrogen atoms were refined anisotropically and hydrogen atoms were positioned with idealised geometry, with the exception of those bound to heteroatoms, the positions of which were located using peaks in the Fourier difference map. The displacement parameters of the hydrogen atoms were constrained using a riding model with *U*_iso_ set to be an appropriate multiple of the *U*_eq_ value of the parent atom.

**Table 1:** Crystal data and structural refinement details for ethyl azocinnamate (I–IV)

	I	II	III	IV
Empirical formula	C_22_H_22_N_2_O_4_	C_22_H_22_N_2_O_4_	C_22_H_22_N_2_O_4_	C_22_H_22_N_2_O_4_
Formula weight	378.41	378.41	378.41	378.41
Crystal system	Triclinic	Orthorhombic	Monoclinic	Monoclinic
Space group	*P*1̄	*P*2_1_2_1_2_1_	*P*2_1_/c	*P*2_1_
*a*/Å	5.9187(2)	3.91720(10)	13.4185(5)	8.8556(2)
*b*/Å	8.0533(3)	9.6395(2)	9.8231(4)	10.0594(3)
*c*/Å	10.5862(3)	50.1648(11)	7.2136(3)	21.7173(6)
*α*/°	99.782(3)	90	90	90
*β*/°	96.732(3)	90	90.961(4)	98.001(3)
*γ*/°	102.517(3)	90	90	90
Volume/Å^3^	479.17(3)	1894.22(7)	950.70(7)	1915.79(9)
*Z*	1	4	2	4
*Z*′	0.5	1	0.5	2
*ρ*_calc_ g cm^−3^	1.311	1.327	1.322	1.312
*μ*/mm^−1^	0.742	0.751	0.748	0.742
*F*(000)	200.0	800.0	400.0	800.0
Crystal size/mm^3^	0.21 × 0.11 × 0.09	0.32 × 0.05 × 0.02	0.14 × 0.07 × 0.01	0.29 × 0.08 × 0.01
Radiation	CuKα (*λ* = 1.54184 Å)	CuKα (*λ* = 1.54184 Å)	CuKα (*λ* = 1.54184 Å)	CuKα (*λ* = 1.54184 Å)
2*Θ* range for data collection/°	8.586 to 154.632	7.048 to 147.28	6.588 to 152.874	4.108 to 154.092
Index ranges	−7 ≤ *h* ≤ 7, −10 ≤ *k* ≤ 10, −13 ≤ *l* ≤ 12	−1 ≤ *h* ≤ 4, −11 ≤ *k* ≤ 11, −62 ≤ *l* ≤ 60	−16 ≤ *h* ≤ 16, −12 ≤ *k* ≤ 11, −8 ≤ *l* ≤ 8	−10 ≤ *h* ≤ 9, −12 ≤ *k* ≤ 12, −26 ≤ *l* ≤ 27
Reflections collected	9088	8816	8654	22 630
Independent reflections	1892 [*R*_int_ = 0.0282, *R*_sigma_ = 0.0200]	3665 [*R*_int_ = 0.0250, *R*_sigma_ = 0.0338]	1866 [*R*_int_ = 0.0295, *R*_sigma_ = 0.0269]	7157 [*R*_int_ = 0.0405, *R*_sigma_ = 0.0422]
Data/restraints/parameters	1892/0/129	3665/0/255	1866/0/128	7157/1/509
Goodness-of-fit on *F*^2^	1.058	1.044	1.067	1.031
Final *R* indexes [*I* ≥ 2*σ*(*I*)]	*R*_1_ = 0.0362, w*R*_2_ = 0.1011	*R*_1_ = 0.0356, w*R*_2_ = 0.0915	*R*_1_ = 0.0386, w*R*_2_ = 0.0977	*R*_1_ = 0.0378, w*R*_2_ = 0.0980
Final *R* indexes [all data]	*R*_1_ = 0.0404, w*R*_2_ = 0.1057	*R*_1_ = 0.0398, w*R*_2_ = 0.0942	*R*_1_ = 0.0455, w*R*_2_ = 0.1032	*R*_1_ = 0.0420, w*R*_2_ = 0.1006
Largest diff. peak/hole/e Å^−3^	0.24/−0.18	0.29/−0.14	0.22/−0.17	0.35/−0.19
Flack parameter	n/a	−0.04(13)	n/a	0.39(9)

## Results and discussion

**Fig. 3:**
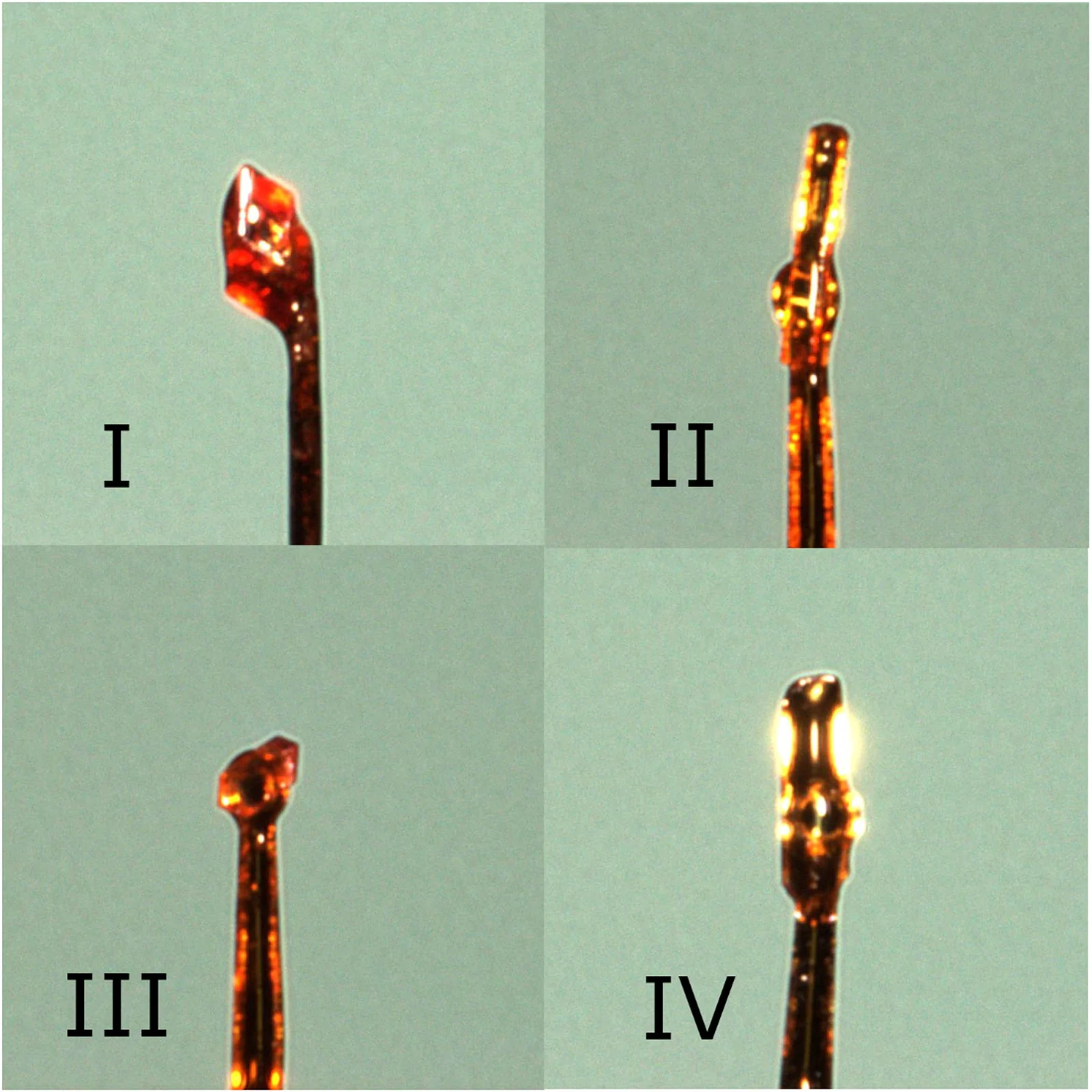
The crystals of I–IV for which data were collected mounted on the same *MiTeGen* MicroMount with a 50 μm aperture.

Crystals of polymorphs I, II and III of ethyl azocinnamate grow concomitantly from ethyl acetate solutions and the representative structures reported in this work were grown in this manner. Similar concomitant mixtures can also be grown from acetonitrile solutions. Each of these polymorphs forms a different crystal habit and were easily distinguishable by eye (Fig. 3). Crystals of I form as red prisms, II as yellow planks and III as hexagonal orange plates. Each crystallisation experiment produced predominantly forms I and II, at the bottom and on the walls of the crystallisation vessel respectively, with a handful of individual crystals of III forming among those of II.

Crystals of IV formed as elongated yellow plates and were more anhedral than the other forms with more rounded corners. This polymorph was observed to be very flexible with many of the crystals appearing curved (see SI). Smaller examples of these crystals can be bent under very little mechanical strain, returning to their original shape once this stimulus is removed, indicative of elasticity in this form, a phenomenon that has been observed in crystals of other azo dyes.^15^ Given the concomitant formation of the various forms it was not feasible to prepare samples of one form suitable for melting point analysis.

In terms of their molecular structure, the four polymorphs exhibited one of two basic conformations (Fig. 1), which are differentiated by their point group symmetry and referred to here as the *C*_2h_-conformer (I and III) or the *C*_s_-conformer (II and IV). In those structures exhibiting the *C*_2h_-conformer, the space group is centrosymmetric and the molecules lie on a centre of inversion such that *Z*′ = 0.5. Molecules of the *C*_s_-conformer lack the inversion centre observed in the *C*_2h_-conformer and are not perfectly planar in the crystal structure, hence they crystallise with integer *Z*′ values. Interestingly, both *C*_s_-conformer structures crystallise in Sohncke space groups despite this conformation being achiral. There appears to be no reason why adjacent *C*_s_-conformer molecules cannot be related by inversion but it seems that if there is to be inversion symmetry in the structure it will manifest as intramolecular symmetry as observed in the *C*_2h_-conformer. Interestingly, the polymorphs that comprise the *C*_2h_-conformer appear darker (red and orange) than those of the *C*_s_-conformer (pale yellow), a case of colour polymorphism.^16,17^

When grouped in pairs of structures in which the molecules adopt either the *C*_2h_- or *C*_s_-conformer, the exact conformations of the molecules can be compared by overlaying them. Generally, variations in conformation can be quantified in terms of the N2–N1–C1–C2, C5–C4–C7–C8 and C7–C8–C9–O2 torsion angles and their equivalents (Table 2). For the most part, the cinnamate moieties of the polymorphs exhibit comparable geometry those of the starting materials, **SM1** and **SM2** (see SI).

Selected torsion angles for polymorphs I–IV

**Table:** 

	I	III
N1–N1–C1–C2/°	5.23(19)	9.67(16)
C5–C4–C7–C8/°	2.82(15)	4.34(17)
C7–C8–C9–O2/°	176.29(11)	167.56(12)

**Table:** 

	II	IV (molecule 1)
N2–N1–C1–C2/°	8.44(19)	11.4(4)
N1–N2–C12–C13/°	4.9(2)	9.3(3)
C5–C4–C7–C8/°	3.9(3)	0.5(3)
C14–C15–C18–C19/°	4.9(3)	0.8(4)
C7–C8–C9–O2/°	172.24(19)	174.7(3)
C18–C19–C20–O4/°	178.1(2)	175.4(3)

**Table:** 

	IV (molecule 2)	
N4–N3–C23–C24/°	12.7(4)	
N3–N4–C34–C35/°	7.0(3)	
C27–C26–C29–C30/°	0.6(3)	
C36–C37–C40–C41/°	17.2(3)	
C29–C30–C31–O6/°	168.0(3)	
C40–C41–C42–O8/°	171.5(3)	

**Fig. 4:**
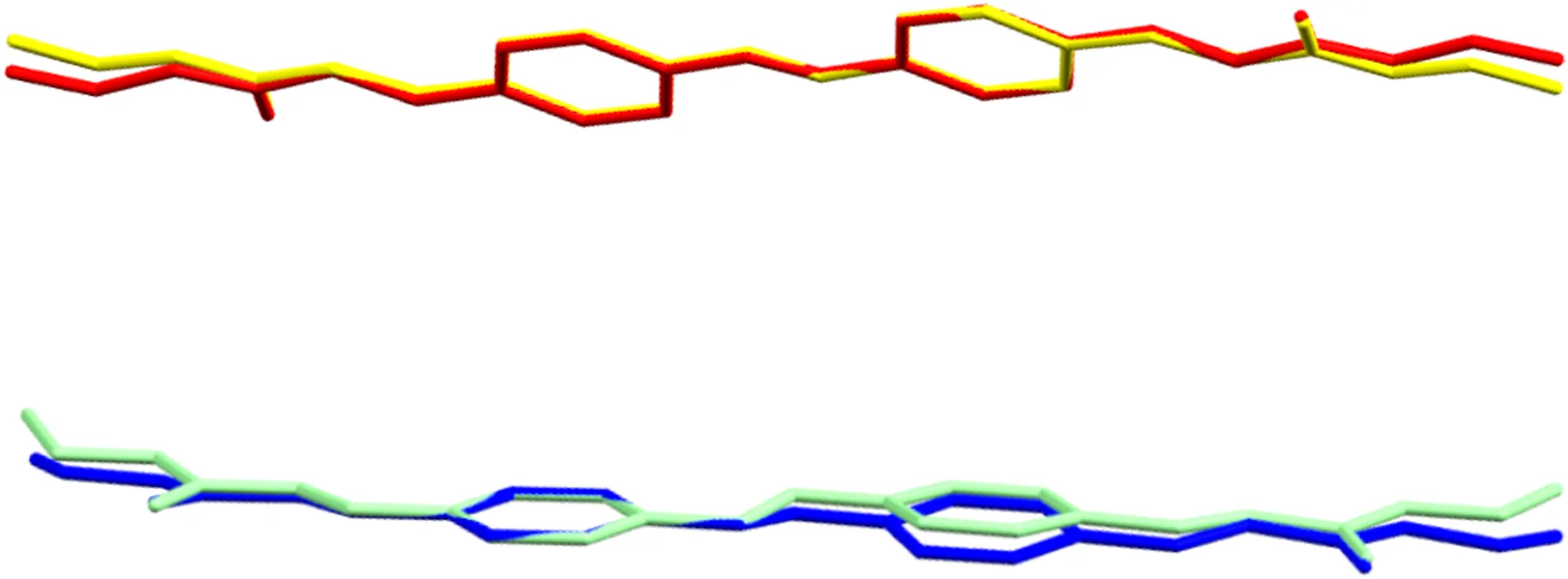
Top: An overlay of the molecules of I (yellow) and III (red); bottom: an overlay of the molecules of II (blue) and IV (green).

To compare and contrast the planarity of the various molecules, RMSDs were calculated with the mean plane defined with respect to all of the non-hydrogen atoms in the given molecule. For I and III, the two polymorphs comprised of the *C*_2h_-conformer, the molecules are essentially planar with RMSD's of 0.089 and 0.114 Å respectively. The conformations vary only slightly (Fig. 4) with the most significant deviation being observed in the C7–C8–C9–O2 torsion angle.

Similar minor variations in conformation are observed between the *C*_s_-conformer molecules of II and molecule 1 in the asymmetric unit of IV. In this case the difference between the two molecules appears to stem from the conformation of the azophenyl moiety as molecule 1 of IV is the most planar of any of the molecules in the four polymorphs, exhibiting the shallowest N–N–C–C torsions.

**Fig. 5:**
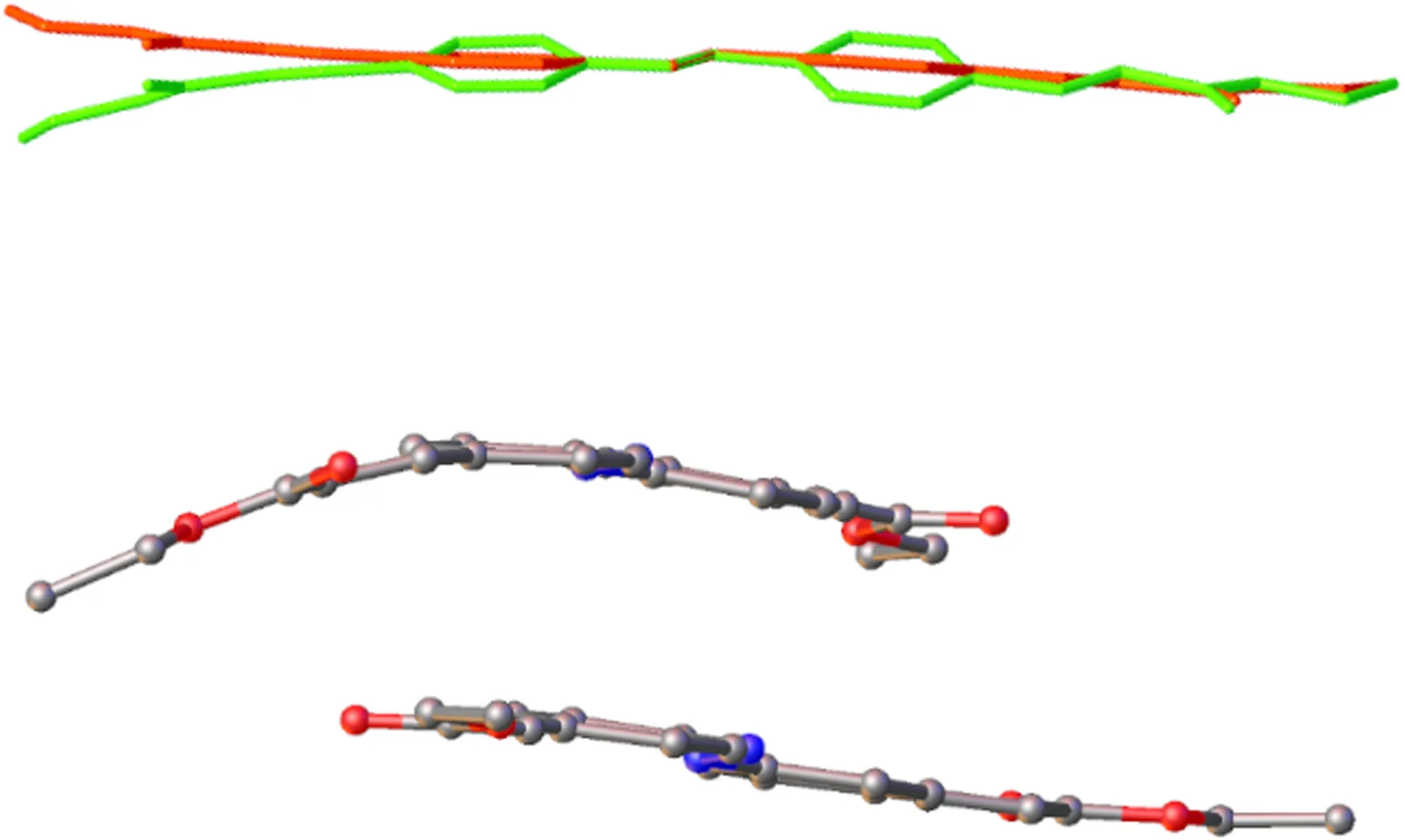
Top: An overlay of the two independent molecules in IV with molecule 1 rendered in orange and molecule 2 in green; bottom: a view of the asymmetric unit of IV highlighting the pronounced bend in molecule 2.

The most striking variation in conformation is observed in the asymmetric unit of IV where the two crystallographically-independent molecules adopt drastically different conformations. Where molecule 1 is essentially planar, with an RMSD of 0.1 Å, molecule 2 exhibits a pronounced bend, which can be attributed to the torsion between one of the phenyl rings and the C=C double bond (C36–C37–C40–C41) differing significantly compared to all of the other molecules across the four forms (Fig. 5). The apparent flexibility of the molecule in IV is curious given the flexibility observed in the crystals of this polymorph.

Moving beyond the individual molecules to consider the interactions between them, for all four polymorphs the nature of the structure-directing interactions is a departure from those observed in **SM1** where the primary amine group allows for the formation of classical hydrogen bonds (see SI). Without similar hydrogen bond donors, weak hydrogen bonding and π-interactions are prevalent in the crystal structures of ethyl azocinnamate (Table 3).

Selected C–H⋯A interactions for I–IV

**Table:** 

	C–H/Å	H⋯A/Å	C⋯A/Å	C–H⋯A/Å
Polymorph I				
C3–H3⋯O1^a^	0.95	2.49	3.3644(15)	153.8
C7–H7⋯O1^a^	0.95	2.56	3.4369(13)	153.6
C11–H11C⋯centroid^b^	0.98	2.65	3.5650(14)	156.4

**Table:** 

Polymorph II				
C10–H10B⋯O4^c^	0.99	2.65	3.594(3)	160.4
C19–H19⋯O1^c^	0.95	2.65	3.536(3)	155.4
C3–H3⋯N1^d^	0.95	2.69	3.541(3)	149.3

**Table:** 

Polymorph III				
C3–H3⋯O1^e^	0.95	2.56	3.4585(16)	158.4
C7–H7⋯O1^e^	0.95	2.76	3.6207(16)	153.4

**Table:** 

Polymorph IV				
C14–H14⋯O1^f^	0.95	2.63	3.573(3)	172.1
C19–H19⋯O1^f^	0.95	2.41	3.245(4)	178.7
C22–H22A⋯O3^g^	0.98	2.66	3.520(4)	147.4
C36–H36⋯O5^h^	0.95	2.75	3.351(4)	122.2
C41–H41⋯O5^h^	0.95	2.44	3.348(3)	142.8
C33–H33B⋯O2^f^	0.98	2.73	3.467(4)	132.5
C44–H44C⋯O7^i^	0.98	2.51	3.411(3)	153.0

a −*X*, 1 − *Y*, −*Z*.

b 1 + *X*, 1 + *Y*, 1 + *Z*.

c 1 − *X*, 1/2 + *Y*, 1/2 − *Z*.

d 2 − *X*, 1/2 + *Y*, 1/2 − *Z*.

e +*X*, 1/2 − *Y*, −1/2 + *Z*.

f 1 − *X*, −1/2 + *Y*, 1 − *Z*.

g −*X*, 1/2 + *Y*, −*Z*.

h 2 − *X*, 1/2 + *Y*, 1 − *Z*.

i 3 − X, −1/2 + *Y*, 2 − *Z*.

**Fig. 6:**
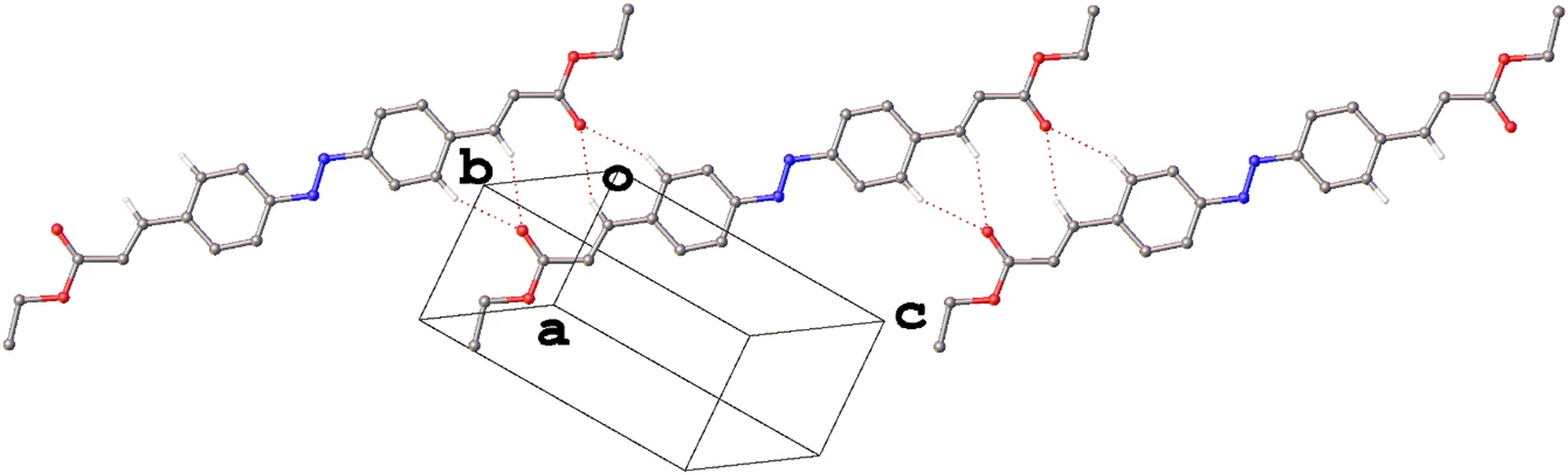
The weak hydrogen bonded chain of molecules along the [11−1] direction in the structure of I. Hydrogen atoms not involved in weak hydrogen bonding have been omitted for clarity.

Weak hydrogen bonding interactions of the type C–H⋯O^18,19^ are observed in all four of the polymorphs. In those comprising the *C*_2h_-conformer, I and III, these interactions form as bifurcated intermolecular bonds with an R^1^_2_(6), 6-membered ring motif.^20^ In I this motif forms across an inversion centre such that each cinnamate moiety is linked by two such interactions to form a chain of molecules along the crystallographic [11−1] direction (Fig. 6). These chains are in turn linked in the [011] direction by Me⋯π interactions the C⋯centroid distances of which are in the range of what would be considered weak CH⋯π interactions.^21,22^

**Fig. 7:**
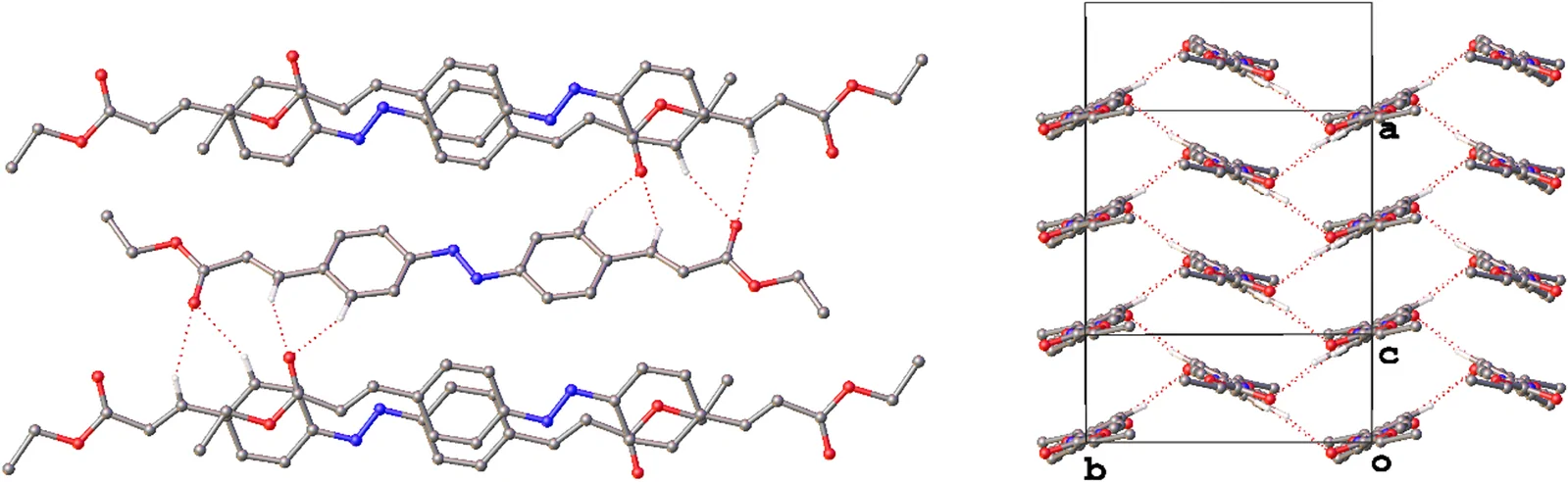
The weak hydrogen bonded network of molecules in the structure of III about one molecule (left) and along [−101] showing the 2D connectivity (right). Hydrogen atoms not involved in weak hydrogen bonding have been omitted for clarity.

Equivalent bifurcated C–H⋯O interactions in the structure of III form not in the reciprocal manner observed in I but such that each interaction links adjacent cinnamate moieties creating chains of molecules in the [001] direction. The inversion centre inherent in the *C*_2h_-conformer allows the identical hydrogen bonded chains at either end of the molecule to link the molecules in the [010] as well creating a 2D network of C–H⋯O interactions with a herringbone motif (Fig. 7). In this case as the molecules in the chain are not essentially coplanar as they are in I, the interactions are slightly longer and more distorted in III.

**Fig. 8:**
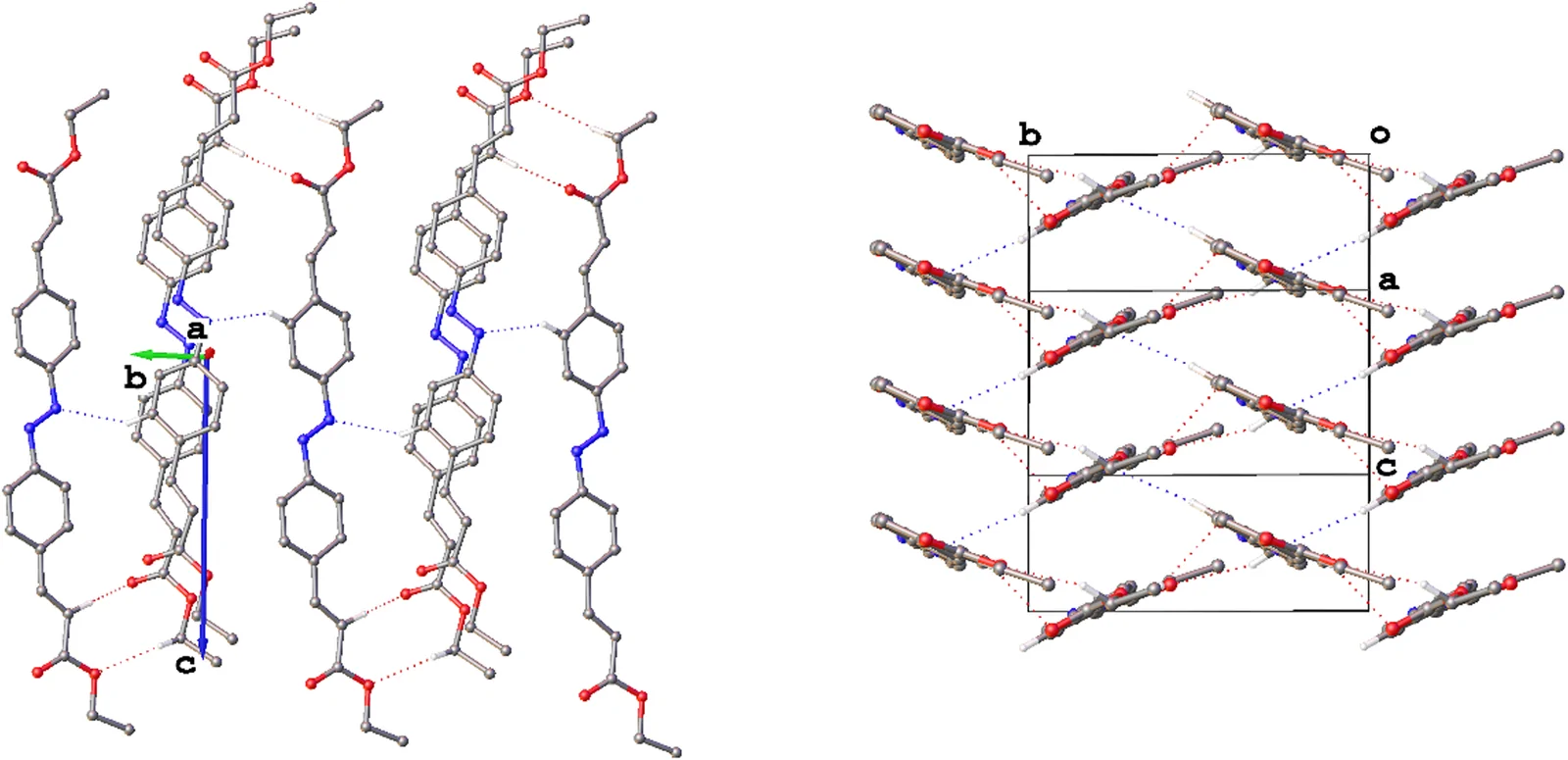
The weak hydrogen bonded network of molecules in the structure of II highlighting the coincident chains of molecules (left) and the herringbone motif of the 2D connectivity (right). Hydrogen atoms not involved in weak hydrogen bonding have been omitted for clarity.

Although the bifurcated weak hydrogen bonds observed in I and III are not present in the structure of II there are still some striking similarities to the structure of III, in particular where their intermolecular interactions are concerned. The molecules in II form chains with a similar alignment to that of III but, as the molecules adopt the *C*_s_-conformation in this case, the cinnamate moieties do not alternate in terms of their orientation as they propagate along the chain. As a result, the weak hydrogen bonds form R^2^_2_(10) ring motifs between the ethyl ester groups (Fig. 8). An additional chain of C–H⋯N interactions is observed along the same [010] direction, intertwining with the chains formed of C–H⋯O interactions. The linking of these chains creates a 2D network with the familiar herringbone motif similar to that observed in III.

**Fig. 9:**
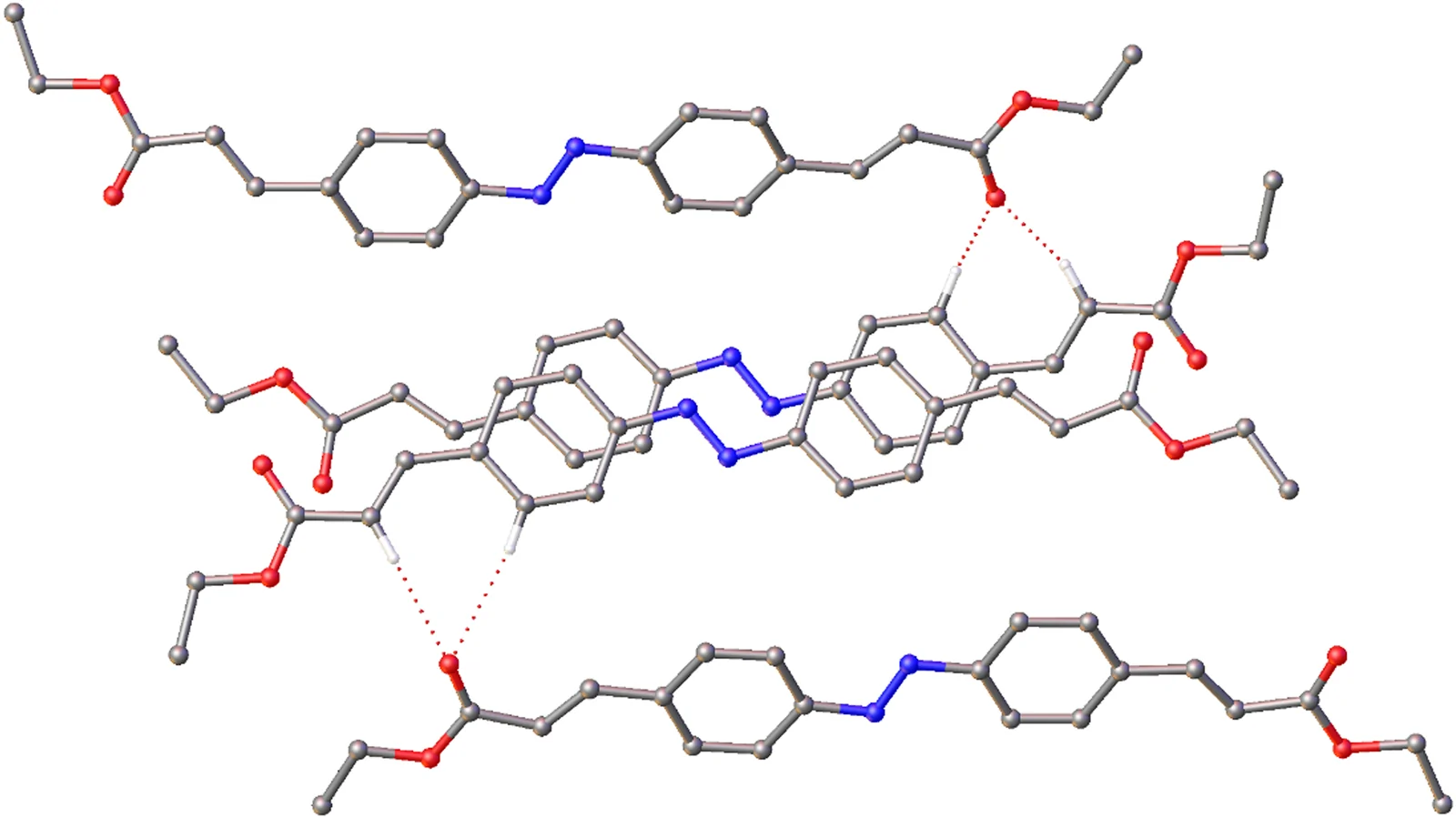
The R^1^_2_(7) weak hydrogen bonding motifs in the structure of IV. Hydrogen atoms not involved in weak hydrogen bonding have been omitted for clarity.

As might be expected given the asymmetric unit comprising two molecules, the network of intermolecular interactions observed in the structure of IV are more complex than those of the other three polymorphs. Bifurcated weak hydrogen bonding interactions are again observed but involving different donor atoms than those of I and III forming R^1^_2_(7) ring motifs in this instance (Fig. 9). One of these rings in particular appears fairly distorted and, as there are many examples of metastable structures with *Z*′ > 1, this is not unexpected.^23,24^

**Fig. 10:**
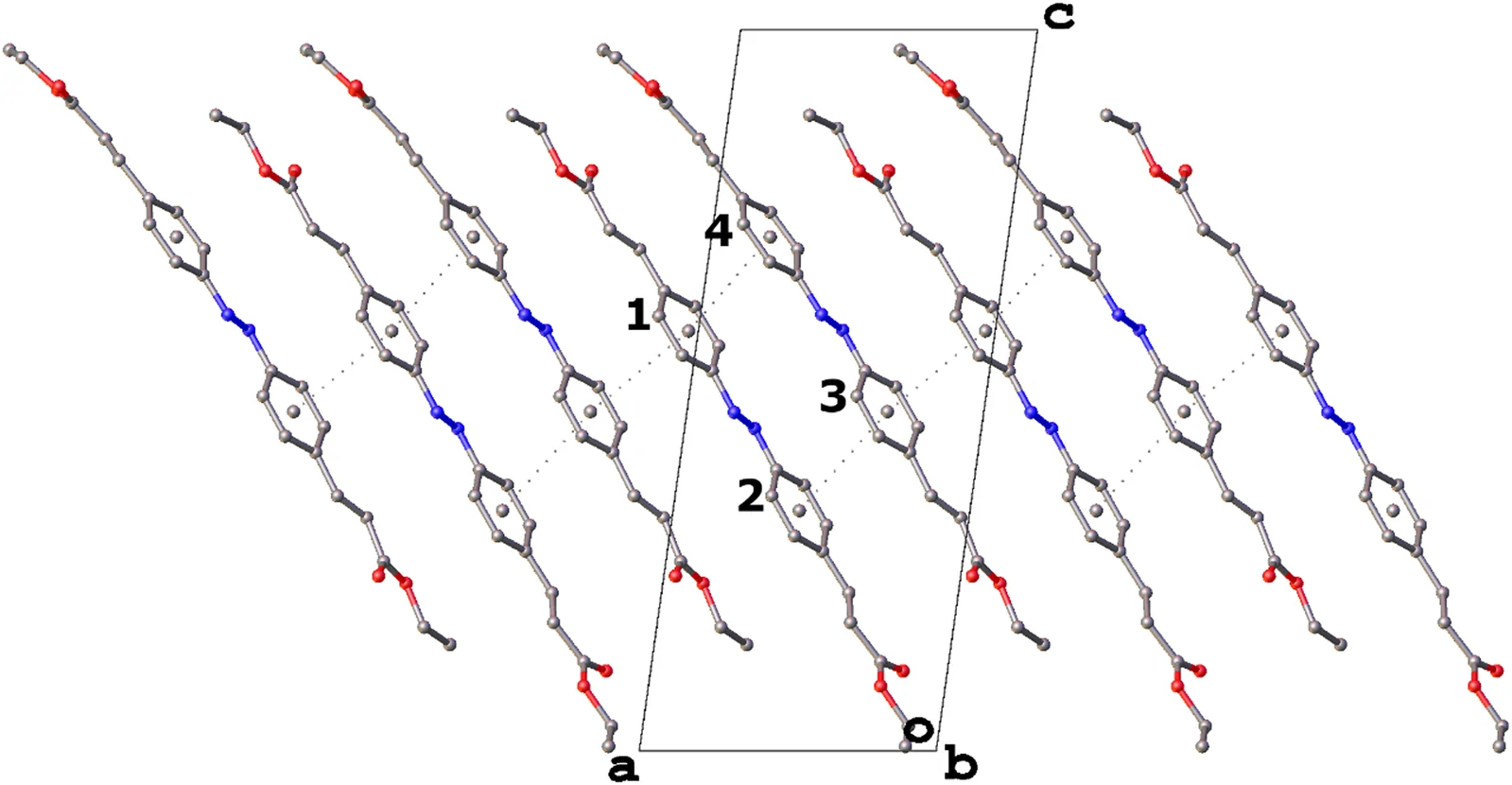
The chain of π⋯π interactions along the [100] direction in the structure of IV with ring numbering. Hydrogen atoms not involved in weak hydrogen bonding have been omitted for clarity.

What is unique about IV with respect to I–III is the presence and propensity of π⋯π interactions in the structure. Though there is one π⋯π interaction with a ring centroid⋯ring centroid distance just below the accepted 4 Å limit in III,^25,26^ there are three such interactions in the structure of IV (Table 4). This results in the formation of stacks of four rings and a continuous chain of π⋯π interactions along [100] direction (Fig. 10).

Ring centroid⋯ring centroid distances between phenyl rings in I–IV (<4 Å)

**Table:** 

	Ring centroid⋯ring centroid/Å
Polymorph III	
Ring1⋯ring1^a^	3.9489(11)

**Table:** 

Polymorph IV	
Ring1⋯ring4	3.9520(16)
Ring2⋯ring3	3.7181(15)
Ring1⋯ring4^b^	3.7866(16)

a 1 − *X*, 1 − *Y*, 1 − *Z*.

b −1 + *X*, +*Y*, +*Z*.

These π⋯π interactions link the bifurcated C–H⋯O interactions in the [010] direction to form the layers in the (001) plane in IV. Additionally, there are also Me⋯O type interactions between these layers that are not apparent in any of the other forms.

The fact that π⋯π interactions are so prominent in the structure of IV can go some way to rationalising the elastic nature of the crystals themselves. Recent work by Akutagawa *et al.* on dihaloanthracenes links π⋯π interactions to the mechanical response of the individual crystals.^27^ The crystals they studied that exhibited flexibility were found to bend in the direction in which columns of molecules formed of π⋯π interactions propagated.

**Fig. 11:**
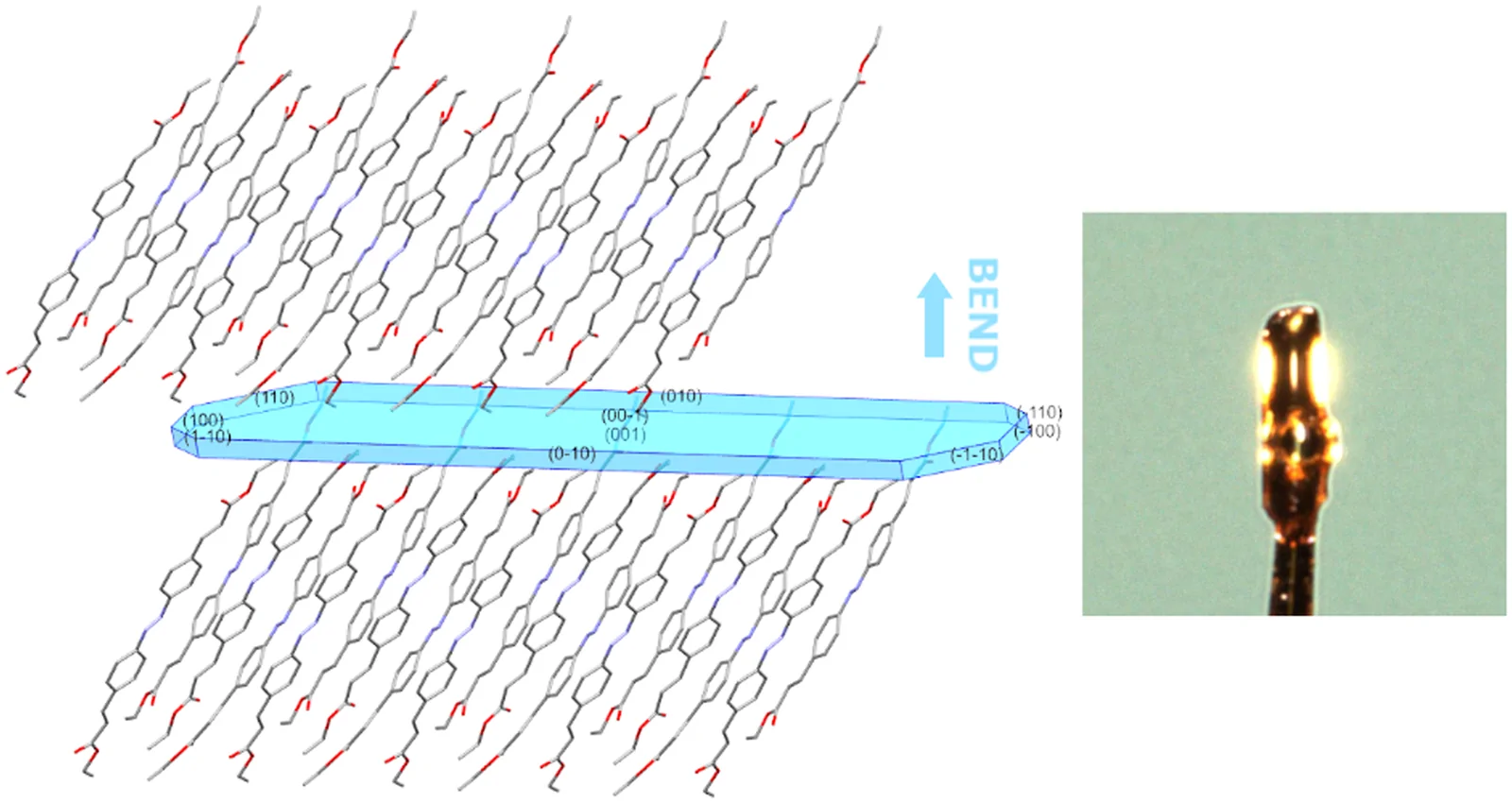
The packing in the structure of IV relative to the crystal for which data were collected. Hydrogen atoms have been omitted for clarity.

Crystals of IV could be bent when mechanical force was applied to the face indexed as (100) in the crystal for which data were collected. By superimposing the exact morphology of this crystal and the structure, it can be seen that the π⋯π interactions do indeed form continuous columns in this direction with slip planes between them,^15^ which would seem to support the assertion that these interactions are key to the flexibility observed in crystals that form these columns (Fig. 11).

**Fig. 12:**
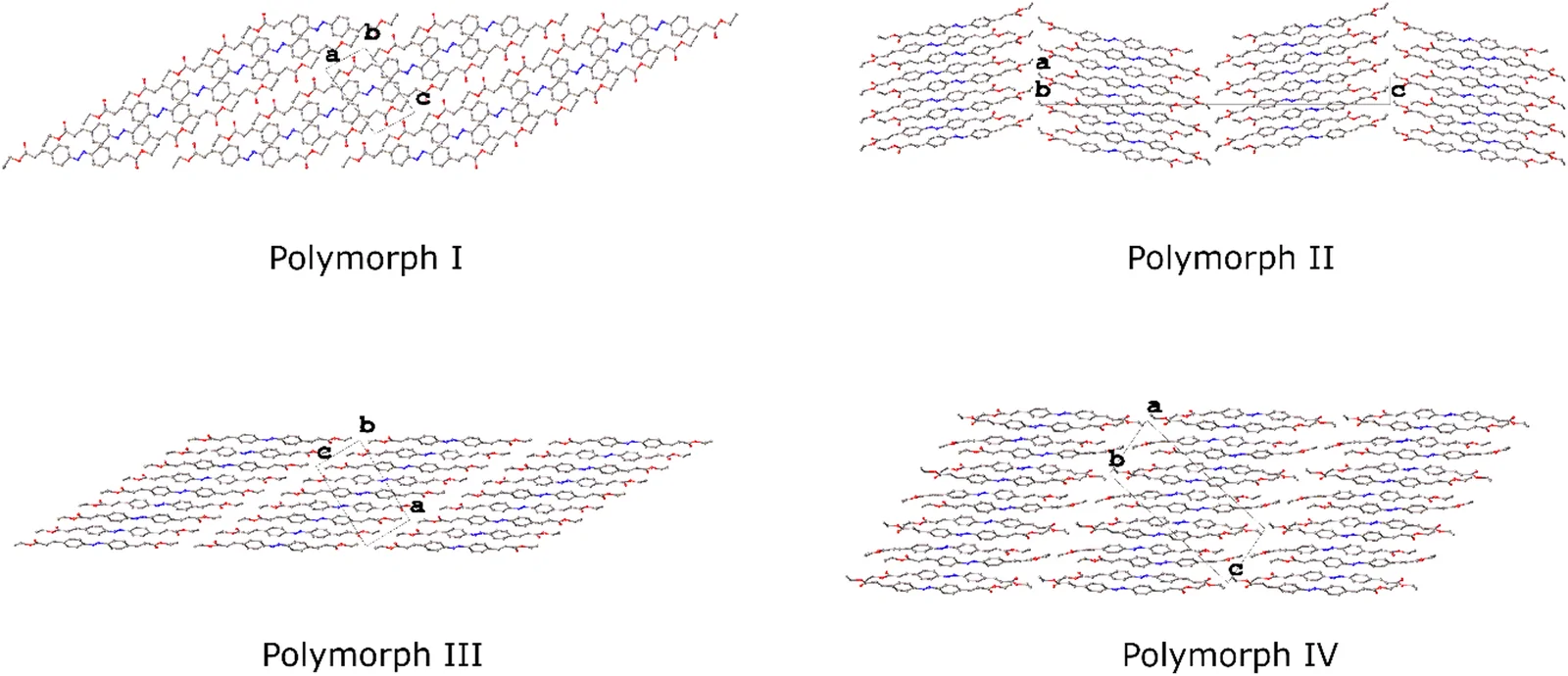
Packing diagrams for I–IV viewed along the [010] direction highlighting the relationship between adjacent layers. Hydrogen atoms have been omitted for clarity.

Analysis of the intermolecular interactions in polymorphs I–IV has established that each form crystallises with a layered structure, and this becomes more evident when considering the packing as a whole (Fig. 12). Where similarities were often seen in the packing within the layers, there are some marked differences in the way these layers pack together across the four structures depending on the symmetry, conformation and the nature of the intermolecular interactions.

Polymorph I appears to be somewhat of an outlier. Though forming in layers, the most obvious structure-directing interactions in the structure, the bifurcated weak hydrogen bonds that form R^1^_2_(6) ring motifs, link the layers together. Considering the various crystal morphologies, II–IV grow as plates or planks with one particularly short dimension in the direction in which the layers stack, whereas I forms as large prisms. The aforementioned interactions in I clearly afford stability and allow the crystals to grow in the direction in which the layers stack in a way that the other polymorphs, which do not exhibit obvious attractive interactions between layers, do not.

Polymorphs II and III share similarities in terms of the packing of individual layers, both in terms of intermolecular interactions and the herringbone arrangement of the 2D layer structure. On its face this might hint that they could be isostructural *C*_2h_- and *C*_s_-conformer structures but the orientation of the layers relative to each other differs drastically in each. In III each layer is related by pure translation symmetry, as is also the case in I and IV, but in II they are related by the symmetry of the 2_1_ screw axis in the [100] direction, which manifests as a herringbone packing arrangement of adjacent layers.

If any two structures are closely related to the point where it might be argued that they are isostructural,^28^III and IV could be those structures. Polymorph IV can be seen as a somewhat distorted *C*_s_-conformer equivalent of III. These are the only two structures to exhibit π⋯π interactions and the orientation of the azophenyl core within layers is similar in both.

**Fig. 13:**
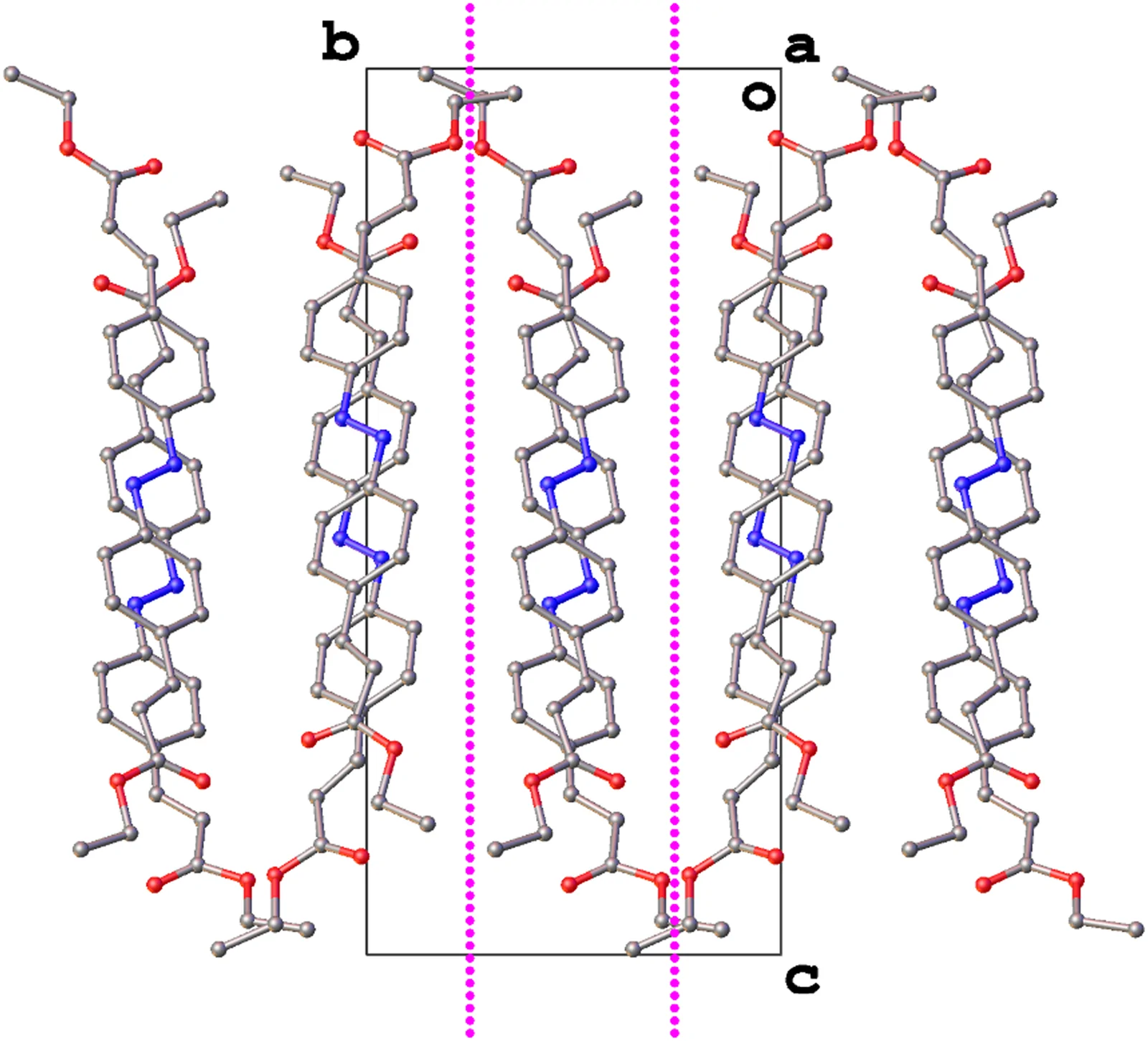
A view of the structure of IV along [100] with approximate glide planes depicted as dotted magenta lines.

This argument is further strengthened considering the symmetry in each structure. Both crystallise in monoclinic space groups: III in *P*2_1_/*c* and IV in *P*2_1_. This alone would not normally be enough of a similarity to imply isostructurality, but there may be more to the structure of IV than is implied by its space group symmetry. Structures like that of IV with *Z*′ = 2 often exhibit approximate symmetry^29,30^ and a closer inspection reveals that this is true of IV (Fig. 13).

The two independent molecules of the asymmetric unit appear related by an approximate inversion broken by the orientation of the azophenyl core and the bend observed in molecule 2. There is also an approximate glide plane at *b* = 0.261 with a translation [100]/2. In this case the symmetry is broken by a translational modulation in the [010] direction. Considering this approximate symmetry, the structure of IV can be described as a distorted *P*2_1_/*c* structure and hence all the more reason to consider IV as being isostructural with III.

## Conclusion

Within a short time after synthesis, four polymorphs of ethyl azocinnamate have been produced using simple crystal growing techniques and their structures determined. All four form as layered structures but with a range of different conformations, intermolecular interactions and packing motifs. The nature of the structures rationalises some of the properties of the crystals such as colour, morphology and flexibility. As such this analysis provides insights into crystal engineering, where linking the structures to their properties in this way can facilitate rational design of bespoke materials for specific applications.

The question remains that, given the speed and ease with which these polymorphs were grown, how many more remain to be discovered? It is easy to imagine for example that forms that consist of a mix of both *C*_2h_- and *C*_s_-conformers may be possible. In addition, given the apparent difference in colour between the two major conformers, could intermediate conformations or mixtures lead to more extensive colour polymorphism as observed in ROY?^16^ With the conclusion of this simple study the next step should be to reach beyond the classical crystal growing techniques used here and apply a high-throughput approach^31^ or utilise melt crystallisation^32^ to fully explore the polymorphic space of this intriguing compound.

## Conflicts of interest

The authors declare no competing financial interests.

## Supplementary Material

CE-028-D5CE01159K-s001

CE-028-D5CE01159K-s002

## Data Availability

Supplementary information (SI): the SI contain figures and tables of refinement details relating the crystal structures of the starting materials and an optical microscopy image showing the flexibility inherent in crystals of one of the polymorphs described in the article. See DOI: https://doi.org/10.1039/d5ce01159k. CCDC 2514195–2 514 200 contain the supplementary crystallographic data for this paper.^33*a*–*f*^
