# Supplementary material for: Four polymorphic structures of a symmetric azo dye
Source: CrystEngComm. 2026 Feb 9;28(11):1781–90. doi: 10.1039/d5ce01159k (PMC13584635; doi:10.1039/d5ce01159k)
Supplement: Supplementary file 1 [file CE-028-D5CE01159K-s001.pdf]

This journal is © The Royal Society of Chemistry 2026

Supplementary Material

Four Polymorphic Structures of a Symmetric Azo Dye

Max T. Hill[1,2], Mark Birch-Machin[3], Jonathan D. Sellars[1,2], Paul G. Waddell[4]

[1] Biosciences Institute, Faculty of Medical Sciences, Newcastle University, Newcastle upon Tyne, NE1 7RU, UK.
[2] School of Pharmacy, Faculty of Medical Sciences, Newcastle University, Newcastle upon Tyne, NE1 7RU, UK.
[3] Translational and Clinical Research Institute, Faculty of Medical Sciences, Newcastle University, Newcastle upon Tyne, NE1 7RU, UK
[4] School of Natural and Environmental Sciences, Bedson Building, Newcastle University, Newcastle upon Tyne, NE1 7RU, UK.

Corresponding author e-mail: paul.waddell@ncl.ac.uk
ORCID: 0000-0002-7851-7347

Table S1: Crystal data and Structure refinement for **SM1**

Empirical formula	$C_{11}H_{13}NO_2$
Formula weight	191.22
Temperature/K	150.0(2)
Crystal system	orthorhombic
Space group	$Pna2_1$
a/Å	22.6748(7)
b/Å	8.4634(3)
c/Å	5.3937(2)
α/°	90
β/°	90
γ/°	90
Volume/Å^3	1035.08(6)
Z	4
ρ_{calc}g/cm^3	1.227
μ/mm^{-1}	0.687
F(000)	408.0
Crystal size/mm^3	0.27 × 0.03 × 0.02
Radiation	CuKα (λ = 1.54184 Å)
2Θ range for data collection/°	7.798 to 153.328
Index ranges	$-22 \leq h \leq 27$, $-10 \leq k \leq 10$, $-2 \leq l \leq 6$
Reflections collected	3449
Independent reflections	1435 [R_{int} = 0.0305, R_{sigma} = 0.0332]
Data/restraints/parameters	1435/1/134
Goodness-of-fit on F^2	1.050
Final R indexes [I>=2σ (I)]	R_1 = 0.0381, wR_2 = 0.0972
Final R indexes [all data]	R_1 = 0.0431, wR_2 = 0.1009
Largest diff. peak/hole / e Å^{-3}	0.22/-0.15
Flack parameter	0.2(3)

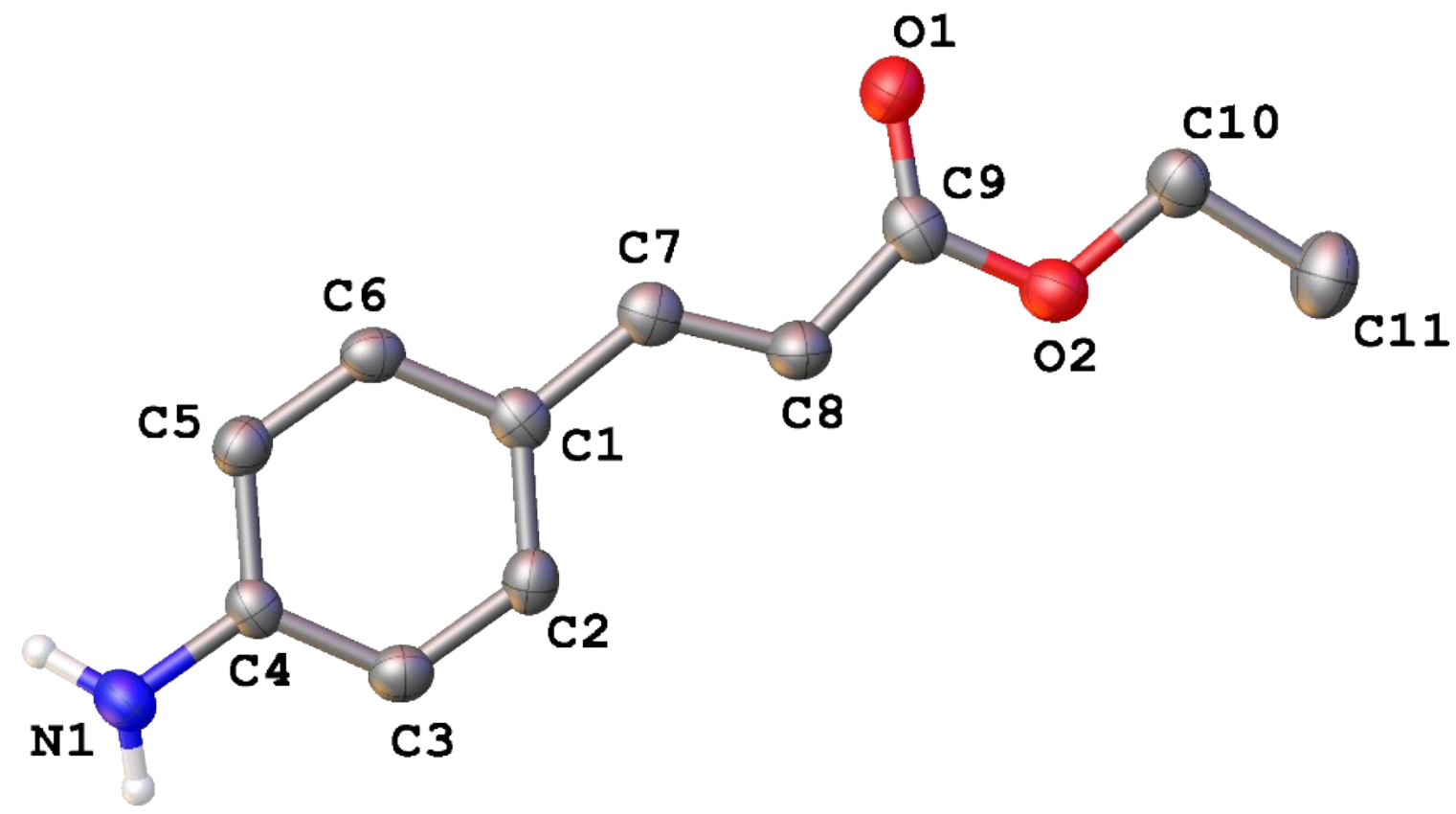

Figure S1: The crystal structure of **SM1** with ellipsoids drawn at the 50% probability level. Hydrogen atoms not bound to heteroatoms have been omitted for clarity.

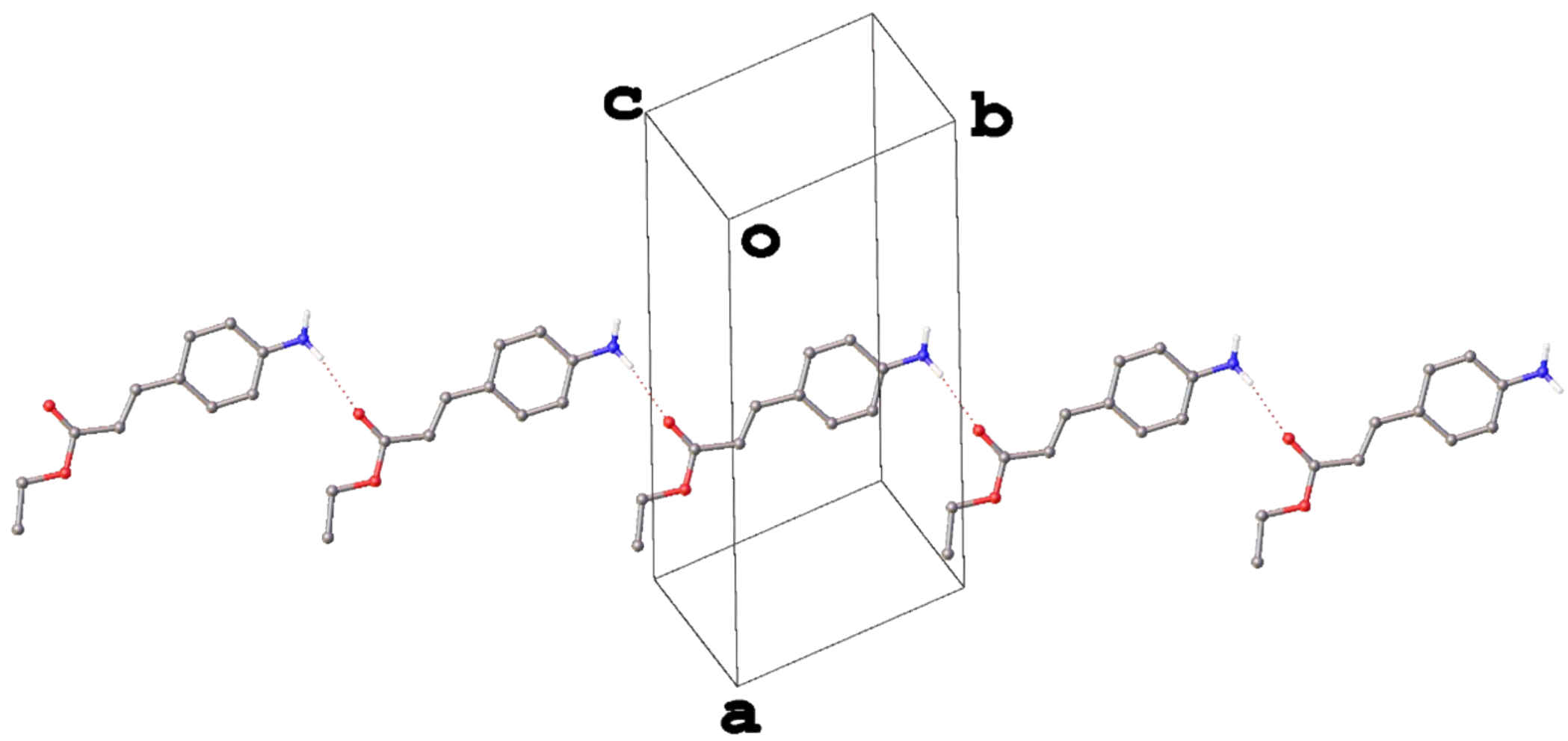

Figure S2: The 1D hydrogen bonding network propagating along the crystallographic [02-1] direction in **SM1** with hydrogen bonds depicted by dashed bonds. Hydrogen atoms not bound to heteroatoms have been omitted for clarity.

Table S2: Crystal data and Structure refinement for **SM2**

Empirical formula	$C_{11}H_{11}NO_4$
Formula weight	221.21
Temperature/K	150.0(2)
Crystal system	triclinic
Space group	*P*-1
a/Å	6.9802(3)
b/Å	8.0029(4)
c/Å	10.9422(7)
α/°	69.905(5)
β/°	72.221(5)
γ/°	68.201(5)
Volume/Å^3	521.80(5)
Z	2
ρ_{calc}g/cm^3	1.408
μ/mm^{-1}	0.914
F(000)	232.0
Crystal size/mm^3	0.15 × 0.11 × 0.03
Radiation	CuKα (λ = 1.54184 Å)
2Θ range for data collection/°	8.79 to 154.208
Index ranges	-8 ≤ h ≤ 7, -10 ≤ k ≤ 10, -13 ≤ l ≤ 13
Reflections collected	9705
Independent reflections	2050 [R_{int} = 0.0296, R_{sigma} = 0.0260]
Data/restraints/parameters	2050/0/146
Goodness-of-fit on F^2	1.082
Final R indexes [I>=2σ (I)]	R_1 = 0.0386, wR_2 = 0.1099
Final R indexes [all data]	R_1 = 0.0428, wR_2 = 0.1139
Largest diff. peak/hole / e Å^{-3}	0.17/-0.23

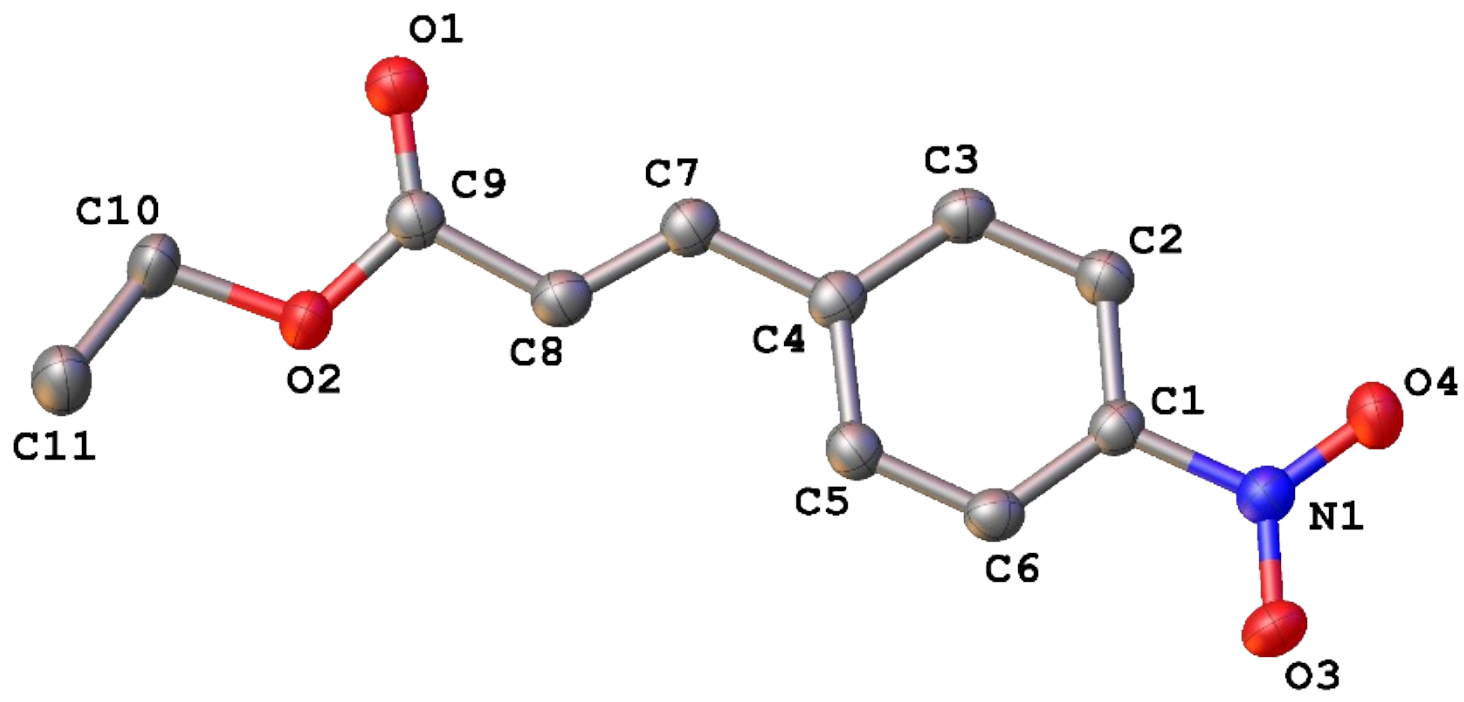

Figure S3: The crystal structure of **SM2** with ellipsoids drawn at the 50% probability level. Hydrogen atoms have been omitted for clarity.

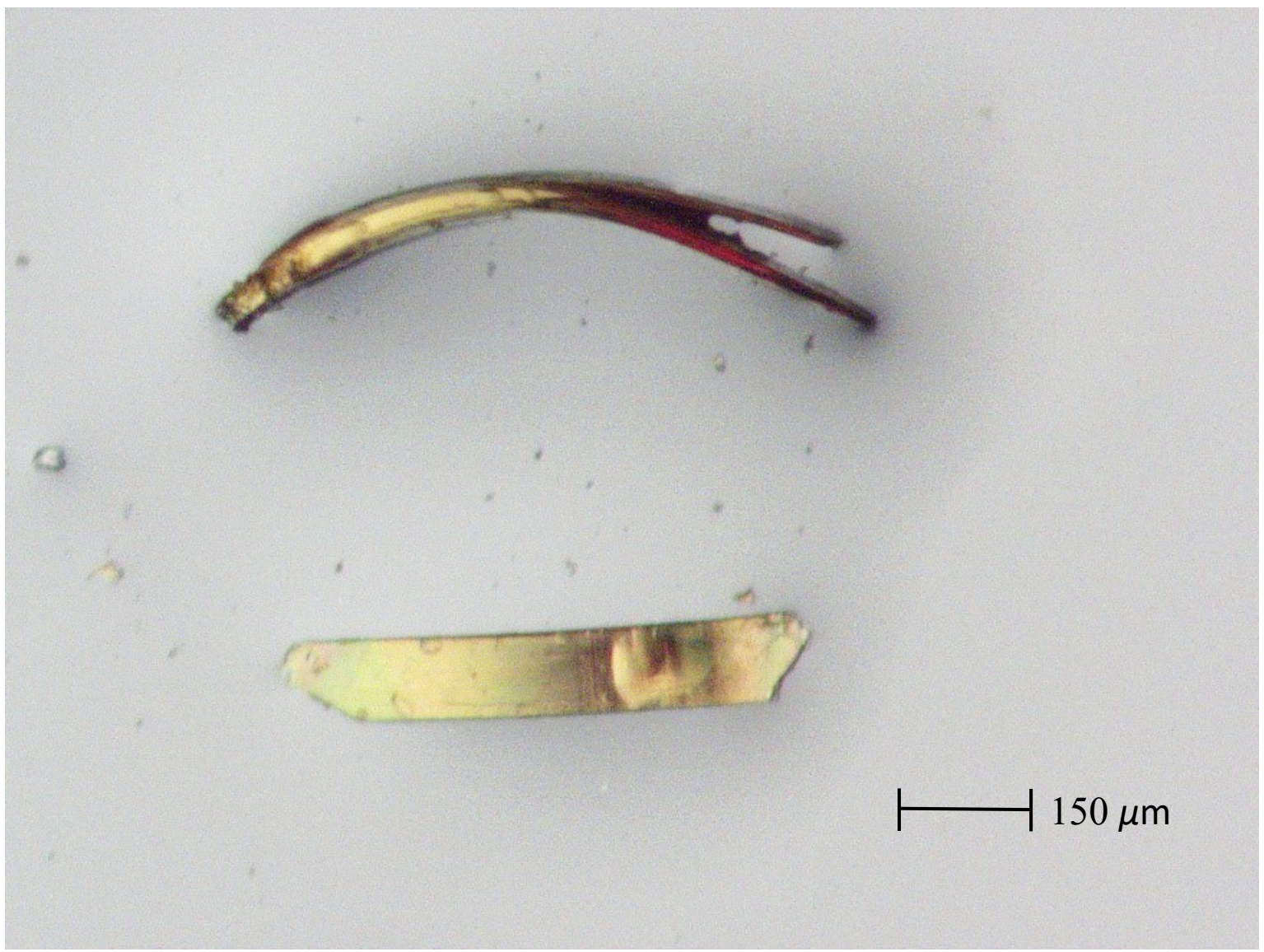

Figure S4: Crystals of polymorph **IV** of ethyl azocinnamate showing the pronounced bend observed in some of the crystals indicative of their inherent flexibility.
